# Poster Session II - A253 HETEROGENEITY OF DISEASE LOCATION IN RANDOMIZED, PLACEBO-CONTROLLED PHARMACEUTICAL TRIALS (RCTS) IN INFLAMMATORY BOWEL DISEASE (IBD)

**DOI:** 10.1093/jcag/gwaf042.253

**Published:** 2026-02-13

**Authors:** A Beamish, S Kumar Vuyyuru, Y Yuan, V Jairath

**Affiliations:** Western University Schulich School of Medicine & Dentistry, London, ON, Canada; London Health Sciences Centre, London, ON, Canada; London Health Sciences Centre, London, ON, Canada; Medicine, Western University, London, ON, Canada

## Abstract

**Background:**

Disease location varies among patients with inflammatory bowel disease (IBD), and may influence the efficacy of advanced therapies. Thus, it is important to understand recruitment pattens based on disease location and its impact of on outcomes in IBD clinical trials.

**Aims:**

We conducted a systematic review to evaluate the distribution of different disease locations and its effect on efficacy of advanced therapies among patients enrolled in IBD clinical trials.

**Methods:**

MEDLINE, Embase, and Cochrane CENTRAL (via OVID) were systematically searched for randomized, placebo-controlled induction trials (RCTs)in patients with IBD, published from 2000 to March 2025. We extracted baseline data on disease location or extent and assessed whether the studies included disease location in the eligibility criteria or considered it as a stratification factor in the randomization. Additionally, we collected data on efficacy outcomes by disease location, if reported.

**Results:**

We identified 367 records and 215 underwent full text review. In total 139 RCTs met inclusion criteria. Among these 71 RCTs included patients with CD (n = 25616) and 68 UC (n = 22959). Among the CD studies, 24.8% (n = 6374) of patients had ileal disease, 36.0% (n = 9210) ileocolonic, 33.1% (n = 8467) colonic and 4.1% (n = 1056) had upper GI involvement. In 2 CD studies, exclusion of upper GI disease location was specified. Among UC studies, only 2.2% (n = 509) patients had proctitis, 49.9% (n = 11455) left sided colitis, and 37.2% (n = 8552) extensive/pancolitis. Although the majority of studies (n = 37) did not include patients with proctitis, only 31 explicitly listed proctitis as an exclusion criterion. No RCT stratified patients based on disease location in randomization; and 27 RCTs (CD:12, UC:15) reported outcomes based on disease locations.

**Conclusions:**

This systematic review provides valuable insights into the distribution of disease location and extent among patients participating with IBD clinical trials. Most CD trials included patients with ileocolonic disease location and patients with proctitis are generally excluded from UC trials. Additionally, disease location generally is not considered as a stratification factor in randomization.

**Funding Agencies:**

SRTP Schulich

